# Recommendations for Ensuring Good Welfare of Horses Used for Industrial Blood, Serum, or Urine Production

**DOI:** 10.3390/ani11051466

**Published:** 2021-05-20

**Authors:** Xavier Manteca Vilanova, Bonnie Beaver, Mette Uldahl, Patricia V. Turner

**Affiliations:** 1 School of Veterinary Science, Universitat Autònoma de Barcelona, 08193 Barcelona, Spain; xavier.manteca@uab.es; 2 College of Veterinary Medicine, Texas A&M University, College Station, TX 77843-4474, USA; bbeaver@cvm.tamu.edu; 3 Vejle Equine Practice, 7120 Vejle, Denmark; mette@vejlehestepraksis.dk; 4 Global Animal Welfare & Training, Charles River, Wilmington, MA 01887, USA; 5 Department of Pathobiology, University of Guelph, Guelph, ON N1G 2W1, Canada

**Keywords:** PMSG, equine chorionic gonadotropin, animal welfare, horse

## Abstract

**Simple Summary:**

Because of their large size, blood, serum, or other substances are often collected from horses for production of biologics and therapeutics used in humans and other animals. There are few international guidelines that provide recommendations for caring for horses kept for these purposes. In this paper, general guidelines are provided to ensure well-being of horses kept for production of biologics.

**Abstract:**

Various pharmaceutical products have been derived from horse blood and urine for over a century. Production of biologics and therapeutics from these samples is a niche industry and often occurs in regions with little regulation or veterinary oversight. To ensure good welfare of horses maintained for these purposes, guidance has been developed to support the industry.

## 1. Introduction

Horses are frequently used for producing medical substances such as hormones, antibodies, immune serum, and other substances. Except for pregnant mare urine and snake antivenom production, there are no international or industry guidelines for much of the work conducted to obtain medical substances from horses. The use of horses for producing therapeutics is likely to continue as long as nonanimal alternatives are unavailable or are significantly more expensive to produce. Thus, the objective of this paper is to establish recommendations to guide industry and ensure good welfare of horses used for producing human and animal biologics and therapeutics.

Horses have been deemed particularly useful for producing therapeutics for human use because of their relatively large blood (or urine) volume, which can be collected repeatedly and extracted for antibody, hormone, or other protein isolation, and because of their general ease in handling and maintenance [1]. As a result, pharmaceutical products have been derived from horses for over a century for use in human diseases and other conditions. The development of equine neutralizing polyclonal antibodies (‘antitoxins’) to treat diphtheria and tetanus was first described in 1890 by von Behring and Kitasato, and equine antibodies were first used successfully to treat a child sick with diphtheria in 1891 [2]. Prior to the use of equine hyperimmune serum there were no effective treatments for the condition, caused by an exotoxin produced by the bacterium *Corynebacterium diphtheriae*, and it was the leading cause of death in children up to the age of 14 [2,3]. By 1895, facilities for largescale production of diphtheria antitoxin from horses had been established by pharmaceutical companies in Germany and were soon to be established in other countries [4]. Tetanus, caused by a toxin produced by the bacterium *Clostridium tetani*, was a similarly devastating and untreatable disease of horses and humans until the tetanus antitoxin (derived from horses) became widely available [5]. 

Given the tremendous success of equine-derived therapies against two particularly devastating conditions, neutralizing antibodies from horse serum were subsequently developed for passive immunization or therapeutic treatment of humans against a wide variety of diseases or conditions, including botulism (*Clostridium botulinum*), gas gangrene (*C. novyi, C. perfringens*, and *C. septicum*), tularemia (*Francisella tularensis*), *Streptococcus pneumoniae*, *Hemophilus influenzae*, meningitis (*Meningococcus* spp.), anthrax (*Bacillus anthracis*), and endotoxim (lipopolysaccharide) [4]. Equine neutralizing antibodies are still used today for passive immunization of humans following rabies virus exposure in parts of the world [6,7,8] and recently have been recommended for treatment of Ebola virus [9], as well as Junin virus infections, the causative agent of Argentine haemorrhagic fever [10]. Equine hyperimmune serum is used as antivenom to treat poisonous bites or stings from various snakes, spiders, jellyfish, stonefish, and scorpions [11]. Horse anti-thymocyte globulin is also used as a first line therapy to treat acquired aplastic anemia, a severe immune-mediated hematopoietic and stem cell precursor disorder that results in pancytopenia [12,13]. More recently, equine hyperimmune serum has been advocated for treating SARS-CoV2-infected patients [14].

In addition to these uses for immunotherapy, large volumes of blood are collected from pregnant horses for production of equine chorionic gonadotropin (eCG, also known as pregnant mare serum gonadotropin or PMSG) for managing fertility in pigs, cattle, small ruminants and other animals destined for human meat consumption [1], and large volumes of urine are collected from pregnant mares to extract estrogen for treating menopausal symptoms in women [15]. In both industries, mares may be intentionally bred to extract hormones that would not otherwise be available in such high concentrations in nonpregnant animals.

## 2. Existing Guidance for the Care of Horses Used for Industrial Blood or Urine Production

Except for pregnant mare urine collection and snake antivenom production, there are no international or industry guidelines for much of the work conducted to produce and extract therapeutic substances from horses, including guidance for oversight, care, and well-being of the horses as well as any foals produced as a result of pregnancy [1]. 

The World Health Organization has produced general guidelines for snake antivenom production; however, the emphasis of the document is on the safety of substances being produced for use in human use rather than on animal welfare [16]. In the course of producing polyclonal antibodies, horses receive multiple injections, for example, 50–200 uL/site over multiple sites [16], and low-grade fevers, abscesses, and local injection site inflammation may result because of the necessary use of adjuvants, in addition to direct side effects from venom injections [17]. More refined adjuvants might reduce these side effects, but because these are low volume industries there is no current incentive to implement refinements [18]. For other horse serum industries, such as diphtheria antitoxin production, development of potent, efficacious, and widely used vaccines has led to a decreased need for antitoxin and a marked decline in global availability of the product. Some diphtheria antitoxin is still made in Russia, Brazil, and India, but the quality is variable, and it is difficult to import into the USA and the EU. This resulted in the tragic deaths of two children suffering from diphtheria in 2017 in the EU [19]. The global shortage triggered a search for and discovery of a nonanimal treatment for diphtheria toxemia that uses recombinant human antibodies [20]. Hopefully, these will gain widespread acceptance as a replacement therapy for equine diphtheria antibodies. 

The use of pregnant mares for urine collection and estrogen production has received animal activist attention in the past because of concerns about insufficient attention to horse well-being [15]. Subsequently, the industry revised practices and expectations for farms managing horses, and pregnant mare urine (PMU) production is currently overseen by the Equine Ranching Advisory Board (ERAB) in Canada. The ERAB, which includes a veterinary behaviorist and veterinary specialists from the American Association of Equine Practitioners (AAEP), has worked together with the Manitoba and Saskatchewan provincial governments in Canada to develop a recommended industry code of practice for the care and handling of horses on PMU ranches [21]. The code sets forth requirements for veterinary care of horses and foals as well as general expectations for maintenance of facilities and management of animals. Adherence to the code and participation in periodic audits are mandatory for participating horse ranches [22]. The industry is reviewed regularly by the Canadian Veterinary Medical Association, the American Association of Equine Practitioners [23], and the American Veterinary Medical Association [24], amongst other groups. This partnership between industry, government, and relevant veterinary associations could serve as a model for how oversight could be managed for other industry sectors that use horse blood or serum for therapeutic products.

In general, Western society, and in particular, veterinary practitioners, remain largely unaware of the use of horses for extraction of eCG or other substances. These are niche industries involving relatively small numbers of horses (tens of thousands) compared to the millions of horses that exist within the multi-billion-dollar global equine industry (https://www.equinebusinessassociation.com/equine-industry-statistics/, accessed on 18 May 2021). In the distant past, pharmaceutical companies normalized the use of horses for serum collection by releasing movies or images of hygienic conditions on farms or in research facilities to the public [5,25]. Attention from animal activist groups has made farms and various industries reluctant to discuss their challenges more broadly. Regardless of the numbers of horses involved, it is essential that guidelines be in place to ensure the care and well-being of these animals that are so essential to human health and animal production industries.

## 3. Animal Welfare and Ethical Considerations

Although there are many definitions for animal welfare, the basic concept of each relates to how well an animal is doing physically and mentally within its environment. The most recognized definition is that of the World Organisation for Animal Health: “animal welfare means the physical and mental state of an animal in relation to the conditions in which it lives and dies” [26]. 

Definitions have evolved over time and across cultures. Originally, the concept of good welfare consisted of good health and the general care that was necessary to maintain it. Animals were expected to have access to food, water, shelter, and health care. More recently, a behavioral component was added to those things considered necessary for good welfare. This highlighted the animal’s need to show species-typical behaviors. Gradually, the concept of animal sentience has been appreciated, and with it, the recognition that the animal’s perceptions really determine the difference between good and bad welfare. As a result, the view of assessing welfare is shifting from the often cited five freedoms to the currently most popular five domains. 

The five freedoms (freedom to express normal behavior and from hunger and thirst; discomfort; pain, injury or disease; fear and distress) are an input-based listing of what animals should have for good welfare [27]. Inputs cover basic things that affect welfare, such as genetics, housing, diet, veterinary care, and training of and handling by the stockperson [28,29,30,31]. Because inputs are relatively easy to measure, they tend to be favored in formal welfare assessments [32]. Alone, however, input-based parameters do not ensure that the animal is receiving good welfare.

Unlike the input-based criteria, outcome-based parameters quantify specific animal responses, such as health, production, behavior, and physiological measurements, and are indicators of how well the management systems (inputs) are working [29,31,33,34]. Although outcome-based parameters are often difficult to measure, they are considered to be better indicators of an animal’s welfare and sentience [35,36,37,38]. They are also more consistent with the revised five domains model, which emphasizes animal perceptions [39]. This model recognizes four physical and functional domains intended to draw attention to areas relevant to welfare assessments. First, the nutritional domain emphasizes the importance of not just food and water but that they be available in adequate amounts and quality. The environmental domain includes factors like shelter quality, environmental temperatures and humidity, outdoor access, environmental enrichments, handling practices, and management practices. The third domain, health, includes all aspects of ensuring good health, elimination of pain, and genetic inputs. Behavior is the fourth domain. It is recognized that not all species-typical behaviors are desirable—fighting for example—but behaviors like grooming and moving around are considered important. The nutrition, environment, health, and behavior domains contribute to the fifth (mental) domain. In each of the five domains, applicable negative and positive factors affecting welfare are compared to ultimately define the animal’s welfare state. The desired goal is to have the positives greatly outweigh the negatives and to eliminate any significantly negative experience or condition altogether.

Interpretations of animal welfare often have an ethical basis, which also changes over time. For some people, animals might be the biological equivalent of a tree (indirect theory). Because the animal would have no moral standing, it could be treated in any manner [40,41]. More commonly, people grant some moral consideration to animals but not so much that they would have full, equal status to humans (direct but unequal theory) [40,41]. The third ethical view (moral equality theory) gives animals and humans equal standing and moral status [40,41].

In consideration of the use of horses for industrial production of natural products and therapeutics, the first question to ask is whether the horse should be used at all? Are there non-animal alternatives available or could they be created? If it is necessary to use the horse, then it is critical they be managed in such a way that outcome-based assessments ensure the animals are receiving the best welfare treatment.

## 4. Husbandry and Care Considerations to Improve Horse Well-Being

### 4.1. Procurement of Horses

Horses bred, purchased, or acquired for the purpose of being used for producing bio-substances, including blood, should be in good mental and physical health. The overall fitness for the intended use should be verified by a thorough veterinary examination of each individual horse. Soundness and good physical health are important because of the environmental and physical stresses the mares will undergo. 

Horses should not exhibit any signs of stress or problem behavior and should be used to handling and trained in a way that obtaining bio-substances, i.e., regular withdrawal of blood, does not create unnecessary stress. The previous history of the horse and the records from the veterinary examination should be known and recorded by the owner.

### 4.2. Identification of Horses

Horses should be individually identified. Hot iron branding is used in several facilities to individually identify horses. However, hot branding is very painful [42] and other methods of individual identification systems should be used, whenever possible. Freeze branding is an alternative to iron branding, as it is less painful [43]. However, freeze branding is more time consuming and requires more equipment than iron branding. In addition, there are several safety issues to be considered when using freeze branding, and therefore, adequate training of personnel is required. Radiofrequency identification (RFID) microchip placement is also a reliable means of identifying individual horses and is considered humane [44].

### 4.3. Transportation of Horses

Horses are typically transported long distances when first procured, when moving from pastures to holding areas for blood collection, and when leaving the operation for slaughter or rehoming. Prior to considering transportation, horses must be assessed to ascertain fitness for transport [45]. Unfit horses and mares in the last 10% of their pregnancy must not be transported, except for veterinary care. Stallions and mares with nursing foals must be separated from other animals during transportation. During transport, the horse’s welfare should be a priority, including observing all applicable national and regional regulations addressing the needs of these animals. Under all circumstances, it is recommended to keep transport to a minimum duration and ensure sufficient ventilation and adequate temperature are maintained. 

Adequate rest periods should be part of the plan for transport, including unloading with rest in a stable facility and the provision of food and water at regular intervals (every 4 h as a minimum is recommended). Transport should never exceed 8 h per 24 h [46,47,48,49,50,51].

### 4.4. Feeding

Horses should be fed a wholesome diet of a sufficient nutritional quality and quantity to maintain them in good condition. The physiological demand for production and lactation requires attention to avoid malnutrition. 

Feed should always contain enough grass or roughage to ensure sufficient fiber intake and chewing time of the horse throughout the day and night. Prolonged time without access to grass or roughage should be avoided (ideally, no more than 3–4 h). Free access to clean water should be always ensured [52,53,54,55].

### 4.5. Housing, Social Contact, and Exercise 

The accommodation should be constructed for horses to ensure safety and according to the specific needs of the individual type of horse. At a minimum, horses must be able to lie down, rest in a natural position, turn around, get up unimpeded, and stand in a natural position. The accommodation must also have adequate regulation of noise, light, temperature, and ventilation [45]. Horses should be protected against adverse weather conditions, as well as insects and possible predators. Sufficient shelter of a size to accommodate all horses on the pasture should be available for all weather conditions. Fences and the ground should be well maintained [56,57,58,59,60,61].

When maintained in barns, the space should fit the size of each horse. At all times, horses should be able to lie down easily and rest in a natural position, turn around and get up unimpeded. For this reason, tie stalls are discouraged. Passageways and lying areas should have non-slippery surfaces. Lying areas should be provided with an adequate amount of good-quality bedding to ensure a dry and comfortable resting area.

Social contact with other horses as well as the horse’s innate need for movement and foraging should be observed, as much as possible. Horses should be given daily access to paddocks or pasture for 4–8 h and where possible together with other horses [55,62,63,64,65,66,67].

Turn out on spacious pasture provides the best incentive for horses to move naturally in a steady slow pace while grazing. If the hours of grazing are limited, care should be taken to give the horses daily adequate exercise according to their individual needs. Horses should not be confined in stalls or paddocks without sufficient time and room for movement.

### 4.6. Handling and Training

Horses used to produce blood and urine products are subject to environments and procedures that can be potentially frightening. For this reason, it is important that each animal undergoes early and appropriate habituation to people as well as training to minimize future distress during handling, moving to and from collection sites, application of apparatus, and collection procedures [68,69]. Habituation training should begin as soon as possible after purchased mares arrive at the collection facilities and proceed slowly over several days at a pace appropriate to the learning rate of the individual mare [68,70,71,72]. This training begins with quiet interactions that allow each animal time to investigate new areas and learn to be comfortable with people around and touching them. Desensitization, a technique of introducing potential triggers very slowly, and chaining of simple-toward-complex tasks using positive reinforcement are the two most useful types of training for the various situations these horses will face [72,73]. It is critical that progress be slow enough to not result in an unwanted behavior and that this is done prior to the actual use of the horse in production. Careful introductions to people, places, and happenings will gradually build the animal’s confidence and make long-term handling easier and less dangerous.

### 4.7. Veterinary Attention and Hoof Care

All horses should be observed diligently. Pregnant mares near the end of pregnancy and until two weeks after foaling, horses currently used for production, ill or injured horses, or horses in less than normal body condition should be observed as often as the condition requires and at least once daily. Any horse appearing ill or injured should be given appropriate care without delay and body condition scores should be monitored [74]. If the horse does not respond to such care, or if pain is suspected, veterinary advice should be obtained.

Facilities for temporary separation of ill or injured horses should always be available. All medication and treatment of horses should always be done according to standards of best practice and in a way so that the overall welfare of the horse is never compromised. Records should be kept of veterinary interventions and treatments as well as mortality or euthanasia logs.

For routine health care, a semi-annual veterinary examination is recommended. This should include monitoring dental and endoparasite status as well as overall soundness and health. Vaccination against tetanus and other enzootic diseases present in the given locale is always recommended.

Trimming of the hooves at regular intervals by a trained professional is also recommended to maintain a good and healthy condition [75].

### 4.8. Specific Husbandry Issues Related to eCG Production

Equine chorionic gonadotropin (eCG) is produced from around day 38–40 of gestation, with peak production between day 55 to 70 of gestation. Production of equine chorionic gonadotropin continues until about day 110 (range 100 to 140 days) of gestation, when blood collection is discontinued. At this point, some farms allow mares to carry the foal to term and then sell the foals, whereas on other farms the pregnancy is terminated so that the mare can be rebred either through natural mating or artificial insemination for a second blood collection period that year. Abortion is accomplished by injection of abortifacients, such as prostaglandins, or by manually forcing open the cervix and rupturing the fetal membranes [1]. 

Abortion of mares is not needed to produce eCG, and when it is done, its only purpose is to increase the amount of eCG produced per mare and year. Abortion is likely to be distressing and possibly painful for the animals. Thus, based on humane care principles and on ethical grounds, abortions should only be conducted on the advice of a veterinarian and then solely for therapeutic reasons.

### 4.9. Euthanasia

The horse, as a sentient being, deserves to be cared for in a responsible way from birth to death. Euthanasia of horses should always be performed when a horse is suffering and is not responding to treatment or when a horse has a chronic or incurable condition that causes pain or distress. Under no circumstances should a horse be abandoned or left to suffer.

A method of euthanasia or killing for slaughter is only considered acceptable when guaranteed loss of consciousness occurs before cardiac or respiratory arrest. This can be achieved through chemical suppression of the brain or mechanical disruption (e.g., captive bolt, gunshot). When captive bolt or gunshot are used, bleeding should follow soon after. Euthanasia or killing for slaughter should be performed by a veterinarian or a trained professional with appropriate skills [76].

## 5. Recommendations for Collection of Blood

### 5.1. Collection Procedure

General guidelines for blood collection in horses can be found in the World Health Organization (WHO) guidelines [16]. In general, the room or area in which blood will be collected should be sanitized and all tubes, needles, and collection bags prepared. Ideally, animals should be weighed before blood collection to increase accuracy of volume withdrawal estimates. A new, sterile needle should be used for each venipuncture if a venipuncture attempt fails or if the needle becomes dislodged during blood collection. No more than two attempts should be made on each side of the neck. The venipuncture site, generally the external jugular vein in a horse, should be aseptically prepared for blood collection, including clipping, cleaning, and wiping with disinfectant. To minimize stress, animals should be habituated to handling and to the collection area, and at least two compatible horses should be present in the collection area at the same time. A topical anesthetic cream may be used to desensitize the skin during venipuncture. Horses should be monitored carefully during the collection period and for the following hour and over the subsequent 24 h. Blood collection should be terminated in any animal evincing signs of anxiety, stress, or distress during collection, such as sweating, defecating, etc. The entire blood collection procedure for a given horse should not last more than approximately 30 min. It is critical that adequate hemostasis be applied following blood collection. Potential adverse effects of bleeding include hematoma formation from inadequate hemostasis, pain at the collection site usually due to poor bleeding technique or inexperience, or an infection at the blood collection site due to poor technique or dirty equipment.

### 5.2. Considerations for Maximum Volume of Blood That Can Be Obtained

On average, the circulating blood volume of most breeds of adult horses is approximately 75 mL/kg, with hot-blooded horses (e.g., Thoroughbreds) having up to 100 mL/kg and draft breeds having less (65 mL/kg) [77]. For a 500 kg healthy, normoweight nonpregnant horse, the total blood volume is approximately 37.5 L. The circulating blood volume in pregnant, obese, or geriatric animals may be as much as 15% lower than that of nonpregnant, healthy weight animals of the same breed and should be factored into the calculation for total volume removed. No specific guidelines have been developed for blood removal from horses; however, collection of blood is a common research animal procedure, and safe multi-species research animal blood collection guidelines have been developed and are widely used [78]. The recommended multi-species blood volume limits for single and repeat sampling are provided in Table 1 [78]. These suggest that for a single blood draw, a maximum of 10% of the circulating blood volume can be removed without the need to provide supplemental replacement fluids. The minimum volume of blood necessary for production needs should be collected. If >10% blood volume is required, the collected volume can be replaced by 3–fold volume of isotonic fluids (e.g., saline, dextrose, lactated ringer’s solution). It is also important to consider accidental blood loss during sampling, e.g., blood loss outside the designated tube or bag, blood loss from wounds or injuries, etc.

Although one study has demonstrated that larger volumes can be collected from horses without death (up to 25% of circulating blood volume), animals were reported to have significant signs of distress during the blood collection, including tachypnea, tachycardia, neck sweating, urination, and defecation, and heart and respiratory rates remained elevated for several hours after collections were complete [79]. In this study, although many blood components, such as albumin, returned to pre-bleed levels within a few days of the bleed, total protein levels and, in particular, globulin levels required up to 31 days to recover to near pre-bleed levels [79]. This suggests that routine collection of larger blood volumes than indicated in Table 1 may impair immunity of horses, which also could impact their overall health and well-being.

A potentially interesting alternative to bleeding is plasmapheresis, i.e., the separation of plasma from blood cells so that only plasma is extracted from the animal and all blood cells are immediately returned into the animal’s body. Although plasmapheresis may be advantageous in terms of animal welfare, care should be taken not to extract an excessive amount of plasma and additional fluids should be administered to help replace those lost during plasmapheresis. Hygiene and extraction practices would have to be improved to ensure that blood cells remained sterile following initial collection [80]. 

## 6. Conclusions

While horses have been used in the manufacturing process of biologics for many years, their welfare has only recently surfaced as a concern. Until such time as non-animal origins can be found for certain products, use of the horse is likely to continue. The value of these horses, as well as their sentience, makes it important that producers ensure the best welfare using programs such as the one described in this paper.

## Figures and Tables

**Table 1:** Recommended blood volume limits and recovery periods (reprinted with permission and modified from [78]).

Single Sampling *		Repeat Sampling	
% Circulating Blood Volume Removed in A Single Sampling	Approximate Recovery Period	% Circulating Blood Volume Removed in 24 h	Approximate Recovery Period
7.5%	1 week	7.5%	1 week
10%	2 weeks	10–15%	2 weeks
15% *	4 weeks	20%	4 weeks

* For single sampling it is not recommended to remove >15% of the blood volume due to risk of hypovolemic shock.

## Data Availability

Not applicable.
